# Is there a place for human fetal-derived stem cells for cell replacement therapy in Huntington's disease?

**DOI:** 10.1016/j.neuint.2017.01.016

**Published:** 2017-06

**Authors:** Sophie V. Precious, Rike Zietlow, Stephen B. Dunnett, Claire M. Kelly, Anne E. Rosser

**Affiliations:** a Brain Repair Group, Sir Martin Evans Building, School of Biosciences, Cardiff University, Museum Avenue, Cardiff CF10 3AX, UK; b Wales Brain Repair and Intracranial Neurotherapeutics Unit (B.R.A.I.N), School of Medicine, Cardiff University, Cardiff CF14 4XN, UK; c School of Health Sciences, Cardiff Metropolitan University, Western Avenue, Cardiff, CF5 2YB, UK; d MRC Centre for Neuropsychiatric Genetics and Genomics, School of Medicine, Cardiff University, Cardiff CF14 4XN, UK

**Keywords:** Huntington's disease, Stem cell, Neural transplantation, Cell therapy, Human embryonic germ cells, Fetal-derived neural precursors, HD, Huntington's disease, hEG, human embryonic germ cells, PGCs, primordial germ cells, FNPs, fetal-derived neural precursors, MSN, medium spiny striatal neurons, WGE, whole ganglionic eminence, ES, embryonic stem cells, iPS, induced pluripotent stem cells

## Abstract

Huntington's disease (HD) is a neurodegenerative disease that offers an excellent paradigm for cell replacement therapy because of the associated relatively focal cell loss in the striatum. The predominant cells lost in this condition are striatal medium spiny neurons (MSNs). Transplantation of developing MSNs taken from the fetal brain has provided proof of concept that donor MSNs can survive, integrate and bring about a degree of functional recovery in both pre-clinical studies and in a limited number of clinical trials. The scarcity of human fetal tissue, and the logistics of coordinating collection and dissection of tissue with neurosurgical procedures makes the use of fetal tissue for this purpose both complex and limiting. Alternative donor cell sources which are expandable in culture prior to transplantation are currently being sought. Two potential donor cell sources which have received most attention recently are embryonic stem (ES) cells and adult induced pluripotent stem (iPS) cells, both of which can be directed to MSN-like fates, although achieving a genuine MSN fate has proven to be difficult. All potential donor sources have challenges in terms of their clinical application for regenerative medicine, and thus it is important to continue exploring a wide variety of expandable cells. In this review we discuss two less well-reported potential donor cell sources; embryonic germ (EG) cells and fetal neural precursors (FNPs), both are which are fetal-derived and have some properties that could make them useful for regenerative medicine applications.

## Highlights

• Cell replacement therapy for neurodegenerative disease.
• The shortage of primary human fetal tissue that necessitates the search for alternatives.
• Potential of fetal derived neural precursor cells.
• What about embryonic germ cells?

## 1 Introduction

Most neurodegenerative conditions are currently untreatable and, for the majority, treatments able to positively influence the underlying pathogenesis are likely to be a long way off (Bartus and Johnson, 2016, Gribkoff and Kaczmarek, 2016, Wild and Tabrizi, 2014). This makes strategies such as cell replacement therapy attractive, because a condition may be a target for cell replacement as long as there is relatively focal (at least in the early stages) loss of defined groups of neurons. There has been interest over the last couple of decades in treating Huntington's disease (HD) with cell replacement therapy. HD is a slowly progressive condition in which there is relentless deterioration of cognitive, motor and psychiatric faculties over a 20–30year period. Currently there is no available disease-modifying treatment, but it represents a good target for cell replacement therapy as it is a well characterised monogenetic condition in which there is relatively focal loss of medium spiny striatal neurons (MSN) (Rosser and Bachoud-Lévi, 2012). Furthermore, it is anticipated that progress made towards achieving functionally effective grafts in HD will be applicable to other neurodegenerative conditions (Dunnett and Rosser, 2014).

One of the key requirements for cell replacement therapy to be functionally effective is that the donor cells have the capacity to be precisely differentiated into the target cell type, i.e., MSNs for HD (Precious and Rosser, 2012). The most convincing evidence so far that cell replacement can be effective in HD comes from both animal and human studies using donor cells derived from the *whole ganglionic eminence* (WGE) in the fetal brain (Pauly et al., 2012, Mazzocchi-Jones et al., 2009, Döbrössy and Dunnett, 2003). The WGE is the area that will eventually become the adult striatum and is where MSNs are born and develop (Deacon et al., 1994, Olsson et al., 1995, Olsson et al., 1998, Marin et al., 2000, Evans et al., 2012, Straccia et al., 2016). Thus, MSNs differentiating from WGE have been committed to an MSN lineage during the process of normal development. Such cells are currently regarded as the “gold standard” for cell replacement in HD.

Optimal grafts result when transplants are derived from fetal WGE collected during the peak period of MSN neurogenesis (i.e., approximately embryonic day 14 in rat and 8–10 weeks gestation in human) (Dunnett and Rosser, 2011). Transplantation of developing MSNs into the degenerating striatum has been shown to ameliorate motor and cognitive deficits in animal studies, primarily in rats and primates (Schackel et al., 2013, McLeod et al., 2013, Paganini et al., 2014, Yhnell et al., 2016). Such studies have allowed the mechanisms underlying the functional improvement to be explored, and have shown that implanted cells can integrate into the circuitry and make functional synaptic connections, providing that they are of the appropriate phenotype (i.e., destined to become MSNs) and were procured within the appropriate developmental window (Dunnett and Rosser, 2014). Preliminary evidence of functional efficacy in human transplants comes from a seminal French study that reported human fetal-derived graft survival and significant improvements in both motor and cognitive function in three patients over an approximately six-year period (Bachoud-Lévi et al., 2000, Bachoud-Lévi et al., 2006). Enhanced FDG-positron emission tomography signal in the frontal cortex of these individuals suggested that the implanted cells had integrated into the striatal neural circuitry and made functional connections with relevant cortical regions (Gallina et al., 2014). The proof-of-concept provided by this study is encouraging and demonstrates that transplantation of “native” developing MSNs into the damaged striatum can produce functional improvements in at least some patients with HD. Nevertheless, there is still a pressing need to undertake further studies of fetal WGE transplantation both to confirm the ability of transplanted WGE cells to improve function and to identify the parameters necessary to increase the reliability of the process and understand which patients are most likely to benefit.

For the longer term, however, it will be necessary to identify expandable sources of donor cells for clinical application, as primary fetal cells present several challenges: they are scarce (an issue compounded by the fact that bilateral transplants in HD require cells from approximately four fetuses, i.e., eight WGEs); they cannot be stored long-term (thus causing logistical problems for coordinating cell collection, surgery and pathological screening of cells); and they are difficult to standardise. Thus, in addition to continuing primary fetal transplants for the reasons outlined above, it is also important to identify cells that can be expanded in number *in vitro* and stored to facilitate GMP (Good Manufacturing Practice) production, whilst maintaining the capability to generate striatal MSNs.

Expandable sources of cells, including human embryonic stem (ES) and human adult-derived induced pluripotent stem (iPS) cells, which can be directed down neural lineages and specified to the required cell type are reviewed extensively elsewhere (Bachoud-Lévi and Perrier, 2014, Choi et al., 2014, Ross and Akimov, 2014, Chen et al., 2014). Here we discuss the potential of two types of expandable cells derived from human fetal tissue; embryonic germ (EG) cells and fetal neural precursor (FNP) cells, as potential donor cells for cell replacement therapy in HD. The reasons for being interested in these cell sources are first, that human fetal tissue will need to be collected for some time to come in order to supply cells for proof-of-concept and optimisation studies as outlined above, and secondly that both cell types have theoretical advantages over hES and hiPS cells for regenerative medicine applications, as discussed further below.

## 2 Human embryonic germ (hEG) cells

EG cells are derived from primordial germ cells (PGCs) that reside in the gonadal ridge of first trimester embryos. PGCs are induced from pluripotent epiblast cells very early in embryonic development (Ohinata et al., 2005), and continue to express markers of pluripotency such as Oct4, Nanog and SSEA-1 (Pashai et al., 2012). *In vivo*, PGCs are unipotent and destined to give rise exclusively to gametes, however, *in vitro* exposure of mouse PGCs to exogenous fibroblast growth factor (FGF) 2, leukaemia inhibitory factor (LIF) and stem cell factor (SCF) can cause conversion to EG cells, which proliferate rapidly and form colonies similar to those observed when culturing ES cells (Durcova-Hills et al., 2008). EG cells show all the hallmarks of pluripotency, including differentiation into cell types from all three germ layers, as well as formation of teratomas and chimeras (Labosky et al., 1994, Stewart et al., 1994). So far, only a small number of laboratories have reported successful conversion of human PGCs to human EG (hEG) cells using FGF2, LIF and membrane-bound SCF provided through feeder cells (Pan et al., 2005, Turnpenny et al., 2003, Shamblott et al., 1998, Liu et al., 2004). However, most groups report that, unlike their mouse counterparts, hEG cells do not form teratomas and cannot be maintained in culture long-term, due to their high tendency to differentiate rather than preserving pluripotent traits (Turnpenny et al., 2006). Although resistance to pluripotency and indefinite culture presents some challenges, it also presents a theoretical advantage in that neuronal precursors derived from such sources may be less likely to overgrow or form tumours post-transplantation; something that is currently a problem for many hES cell derived donor cells (Master et al., 2007).

**Fig. 1:**
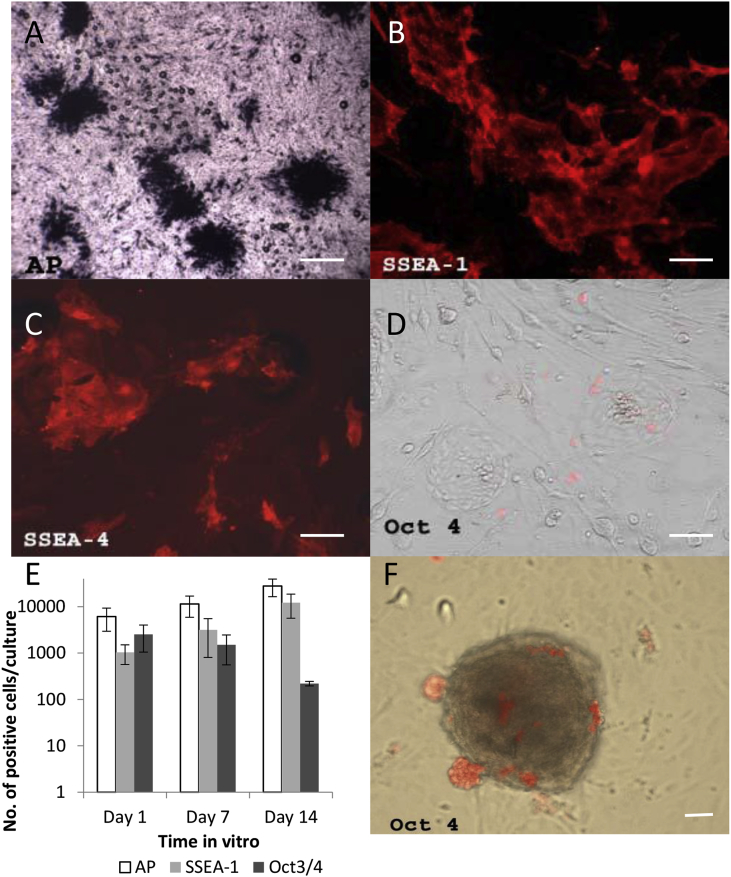
Characterisation of the *in vitro* characteristics of human fetal tissue derived PGCs revealed: A) Alkaline phosphatase positive PGCs (dark blue/black), B) SSEA-1 (red) and C) SSEA-4 (red) but very few Oct4 (red) positive cells, D). Over 14 days in culture the proportion of cells expressing AP and SSEA-1 increased, with AP staining consistently more abundant than SSEA-1 but expression of Oct3/4 fell (E), suggesting that AP staining, whilst convenient, is probably not a reliable PGC marker on its own. When small tissue fragments were present in these cultures, Oct4 positive cells (red) appeared largely to be on the outside of the fragments and formed visible clusters on the surface irrespective of the culture medium used (F). Scale bar = 100 μm. *Abbreviations:* AP – alkaline phosphatase; PGCs – primordial germ cells. (For interpretation of the references to colour in this figure legend, the reader is referred to the web version of this article.)

To explore the possibility that hEG cells could be used for cell replacement applications, we attempted to generate our own hEG cell cultures. Human fetal tissue was collected through the *South Wales Initiative for Transplantation* (SWIFT) program in accordance with the Polkinghorne and Department of Health guidelines, with full ethical committee approval, and under the Cardiff University Human Tissue Act 2004 research licence (Kelly et al., 2011). We harvested gonadal ridges from over 100 human embryos, ranging in age from 6 to 12 weeks gestation, and subjected them to established methods in order to first generate cultures of PGCs. Harvested tissues gave rise to proliferating cells that were positive for alkaline phosphatase (AP), regarded as a marker of pluripotency as well as of PGCs (Fig. 1A). Some also expressed SSEA-1 and SSEA-4 (Fig. 1B and C, respectively), but very few were Oct4 positive (Fig. 1D). Over 14 days in culture the proportion of cells expressing AP and SSEA-1 increased, with AP staining consistently more abundant than SSEA-1 (Fig. 1E). Conversely, expression of Oct4 fell (Fig. 1E), suggesting that AP staining, whilst convenient, is probably not a reliable PGC marker on its own.

Having obtained PGCs in culture, the next step was to convert them to stable EG cell lines. In line with the literature on hEG cell derivation, this proved more difficult. Despite using numerous tissue samples of various ages and exploring numerous different parameters including media components, growth factors, feeder cells, passaging techniques and cell isolation, we failed to convincingly generate hEG cell colonies from any of our PGC cultures.

The manipulations tested are summarised in Table 1 and included:

• ***Growth factors.*** All our cultures contained both FGF2 (based on reports of successful conversion of PGCs to EG cells in both mouse and human (Shamblott et al., 1998, Turnpenny and Hanley, 2007)) and either LIF or oncostatin, another member of the interleukin-6 family that has overlapping activity (Mosley et al., 1996). We also tested: soluble SCF, required for survival and proliferation of PGCs (Tu et al., 2007, Dolci et al., 1991, Godin et al., 1991, Matsui et al., 1991); GDNF, found in female germ cells and needed for germ cell survival in male mice (Miles et al., 2012, Farhi et al., 2010); EGF, which induces proliferation in chick PGCs (Ge et al., 2009); and the nodal signalling molecule activin A, which is critical for maintenance of pluripotency in human cells and stimulates Oct4 transcription in spermatogonial stem cells (Vallier et al., 2005, He et al., 2009). None of these factors, either alone or in combination, led to the appearance of pluripotent hEG cells.
• ***High dose (100 ng/ml) SCF supplementation,*** added to either StemPro 34 medium or standard medium containing 15% fetal calf serum (FCS), as this has been reported to promote colony formation of germ cells in mixed fetal gonadal cultures grown without feeder cells (Tu et al., 2007). We observed that when small tissue fragments were present in these cultures, Oct4 positive cells appeared on the outside of the fragments and formed visible clusters on the surface, irrespective of the culture medium used (Fig. 1F). With continued culture, the somatic cells of the gonadal fragments also attached to the dish and spread out to form a monolayer on which PGCs migrated out. Twenty-four hours after plating, PGC clusters could be removed under a dissection microscope and transferred to feeder-coated plates, where they remained visible for several weeks but did not convert into hEG cell colonies.
• ***Various feeder layers.*** The STO mouse embryonic fibroblasts cell line (ATCC CRL-1503) has been reported as the most successful cell line for this purpose (Turnpenny and Hanley, 2007, Shamblott et al., 1998), but is known to drift with passage, such that sub-clones can vary considerably in their properties, including expression of membrane bound SCF. We used several cloned STO lines, the spontaneously immortalised testicular stromal cell line JK-1 (Kim et al., 2008), and several non-transformed lines of human fetal somatic fibroblast-like cells (He et al., 2007).
• ***PGC purification*.** A practical obstacle to maintaining cultures of PGCs was the high proliferative capacity of somatic gonadal cells. We attempted magnetic bead purification (MACS) using the pluripotency marker CD133, which is expressed in the germline (Gashaw et al., 2007). However, this did not result in a sufficiently pure population of human PGCs that avoided contamination with somatic cells. As germ cells are highly prone to apoptosis *in vitro*, preventing isolation by FACS, we attempted a gentler approach by adapting methods developed for isolating spermatogonial stem cells from adult testis samples using a three-step matrix selection process on plastic, collagen and laminin, followed by culturing on feeder cells to induce conversion to pluripotent stem cells (Conrad et al., 2008). However, PGCs did not form clusters under these conditions.

**Table 1:** Experimental parameters applied to PGC cultures for generation of hEG-derived cell lines.

Base medium^a^	Growth factors added^b^	Dissociation reagents^c^	Feeder layer	Number of embryos	MACS sorted
1	FGF2 (4 ng/ml), LIF (1000U/ml)	Collagenase IV (1 mg/ml)	STO	6	No
1	FGF2 (4 ng/ml), LIF (1000U/ml)	Collagenase IV (1 mg/ml)	STO; SNL; high SCF expressing clonal STO line; 3 human gonadal somatic cell lines	7	No
2	FGF2 (2 ng/ml), LIF (1000U/ml), Activin A (10 ng/ml)	Trypsin/EDTA (0.25%)	STO; SNL	22	Yes
1 & 2	FGF2 (4 ng/ml), LIF (1000U/ml)	Trypsin/EDTA (0.25%)	SNL	4	Yes
3	FGF2 (25 ng/ml), Oncostatin (10 ng/ml), SCF (10 ng/ml)	Trypsin/EDTA (0.25%)	STO	9	No
3 & 2	FGF2 (2 ng/ml), LIF (1000U/ml), plus: Activin A (10 ng/ml), SCF (4 ng/ml) & ROCK inhibitor (10 μM), in the following combinations: A + R, A + S, R + S, A + R + S, Control	Trypsin/EDTA (0.25%)	STO	7	No
2	FGF2 (10 ng/ml), Oncostatin (10 ng/ml), SCF (4 ng/ml)	Trypsin/EDTA (0.25%)	JK-1; STO	8	No
4	FGF2 (10 ng/ml), EGF (20 ng/ml), LIF (1000U/ml), GDNF (10 ng/ml), SCF (100 ng/ml)	Collagenase	None	16	No
4 & 2	FGF2 (10 ng/ml), EGF (20 ng/ml), LIF (1000U/ml), GDNF (10 ng/ml), SCF (100 ng/ml)	Tunica albuginea removed, pulled into fragments in DNAse	None, or STO; Each followed by transfer of PGC clusters onto STO feeder layer	19	No
4	Control, FGF2 (10 ng/ml), GDNF (10 ng/ml), SCF (100 ng/ml), plus combinations: F + G, S + F, S + G, S + F + G	Tunica albuginea removed, pulled into fragments in DNAse	None	3	No

a **Base medium used**: ***(1)*** k.o. DMEM (*Invitrogen*), 15% k.o. serum replacement (*Invitrogen*), 1 mM glutamine (*Invitrogen*), 0.1 mM non-essential amino acids (*Invitrogen*), 0.1 mM β-mercaptoethanol (*Sigma*), 100U/ml penicillin (*Invitrogen*), 100 μg/ml streptomycin (*Invitrogen*), 10 μM forskolin (*Sigma*) (Turnpenny et al., 2003). ***(2)*** DMEM (*Invitrogen*), 15% Fetal calf serum (FCS) (*Hyclone*), 2 mM glutamine, 0.1 mM non-essential amino acids, 0.1 mM β-mercaptoethanol, 1 mM sodium pyruvate (*Invitrogen*), 100U/ml penicillin, 100 μg/ml streptomycin, 10 μg/ml forskolin (Shamblott et al., 1998). ***(3)*** k.o. DMEM, 15% k.o. serum replacement, 1 mM glutamine, 0.1 mM non-essential amino acids, 0.1 mM β-mercaptoethanol, 10 μM forskolin. ***(4)*** Serum-free Media (SFM) (*Invitrogen*), StemPro 34 supplement (*Invitrogen*), 1% FCS, 25 μg/ml hr-insulin (*Sigma*), 200 μg/ml hr-transferrin (*Sigma*), 60 μM putrescine (*Sigma*), 30 nM sodium selenite (*Sigma*), 6 mg/ml d-glucose (*Sigma*), 30 μg/ml pyruvic acid, 1 μl/ml dl-lactic acid (*Sigma*), 5 mg/ml BSA (*Sigma*), 2 mM l-glutamine, 0.1 mM β-mercaptoethanol, 1% minimal essential medium vitamin solution (*Invitrogen*), 0.1 M ascorbic acid (*Sigma*), 10 μg/ml d-Biotin (*Sigma*), 30 ng/ml β-estradiol (*Sigma*), 60 ng/ml progesterone (*Sigma*), 10 μg/ml forskolin (Kanatsu-Shinohara et al., 2003). NB: unless indicated, penicillin and streptomycin were not added to culture medium as they may interfere with maintenance of some stem cell populations.

b Growth factors were obtained from: FGF2 (*R&D Systems*), LIF (*Sigma*), Oncostatin, EGF (*Sigma*), SCF (*Sigma*), GDNF (*Peprotech*), Activin A (*Peprotech*). Control is defined as base medium only with no growth factors added.

c Dissociation reagents were obtained from: Collagenase IV (*Invitrogen*), Trypsin EDTA (*Invitrogen*), ROCK inhibitor (*Merck Chemicals*), DNAse (*Sigma*). Feeder layers used are defined as: STO (mouse embryonic fibroblast line (ATCC CRL-1503); SNL is a LIF-expressing clonal STO cell line; JK-1 is a spontaneously immortalised testicular stromal cell line.

Despite the numerous different manipulations carried out in a large number of fetal samples, and the successful (albeit short-term) culture of PGCs, we were unable to generate any hEG cell lines. We did, however, observe an effect of high SCF concentrations on human PGC adhesion and migration, which could lead to future tools for isolating this highly vulnerable cell type. It seems likely that the factors which can induce de-differentiation of mouse PGCs to a pluripotent state are not sufficient for the human equivalent. This is congruent with the fact that no long-term expandable hEG cell lines have become available to researchers, despite their generation first being reported in 1998 (Shamblott et al., 1998). Fundamental differences between mouse and human gamete formation may further prevent establishment of stable hEG cell lines, the timing and regulation of epigenetic re-programming being obvious candidates.

## 3 Fetal-derived neural precursors (FNPs)

It has long been known that proliferative cells with the capacity to differentiate into cells of a neural lineage (FNPs) can be isolated from the developing fetal WGE, and other brain regions. This was initially shown in rodents (Reynolds et al., 1992) and since then numerous papers have demonstrated similar findings in both rodent and human (Murphy et al., 1990, Murphy et al., 1994, Bartlett et al., 1995, Fricker et al., 1995). There are clear species differences in the responses of FNPs to specific proliferation media, with murine cells tending to proliferate for much longer periods of time than rat and porcine cells (Svendsen et al., 1998, Ciccolini and Svendsen, 1998, Armstrong et al., 2000, Armstrong et al., 2002). In addition, if hFNPs could survive cryopreservation this would ease current practical constraints associated with scheduling the neurosurgery and would also permit at least some standardisation of the cells, which cannot currently be achieved for primary hWGE. Specifically, hWGE can only be reliably held in culture (using media to reduce metabolic processes, ie “hibernation”) for a short period of time (up to 8 days; Hurelbrink et al., 2000). This is not a long enough period of time to permit full quality control of the tissue. Furthermore, FNPs are multipotent, rather than pluripotent, and are lineage restricted. Thus they are less likely to give rise to fast-growing tumours following grafting, in contrast to grafts of pluripotent-derived cells.

A major problem for use of FNPs for regenerative medicine has been the tendency for hFNPs to produce progressively fewer neurons with increasing periods of time in culture. Furthermore, hWGE-derived FNPs that have undergone passaging *in vitro* produce fewer MSN-like neurons and survive poorly following transplantation into rodent HD models (Caldwell et al., 2001, Zietlow et al., 2012). We have previously suggested that the loss of neurogenic potential may be due to loss of positional information as the hFNPs continue to proliferate in the absence of developmental signals to which they would normally have been exposed during development in the embryo (Zietlow et al., 2005). Indeed, PCR analysis of mouse WGE-derived FNPs, compared to primary WGE, shows a dramatic fall in the levels of striatal-specific gene expression (unpublished observations), although in human cells, at least over the first few passages, the expression of striatal markers appeared relatively stable (Martín-Ibáñez et al., 2016). Despite this apparent phenotypic change, short term proliferation of hFNPs still results in a modest increase in cell number (1.6 fold increase in cell number over 10 days in culture), which could have important practical applications (Kelly et al., 2007).

Previous data of ours suggests that, in contrast to transplanted long-term expanded hFNPs, transplants of short-term (10 day) expanded WGE-derived hFNPs retain the ability to survive and integrate into the host brain (Kelly et al., 2007). This study reported that grafts of 10 day expanded hFNPs analysed after 12 weeks survival *in vivo* sent out fibre projections that were target-specific for cells of that origin. Furthermore, the hFNP grafts produced more profuse outgrowths than did primary WGE grafts (Kelly et al., 2007). This data was encouraging in that it suggested that short-term expanded FNPs had a potential to reconnect neural circuitry that is as good, if not better, than that of primary fetal cells, but their low proliferative potential made them less attractive than the highly proliferative pluripotent stem cells. However, recent reports have highlighted that the challenge of directing hES cells to a genuine MSN phenotype capable of ameliorating functional deficits has so far been greater than initially anticipated. There are sporadic reports of limited improvements in small preclinical studies, for example some improvement in rotation following ES cell-derived MSN grafts (Delli Carri et al., 2013), but to date this falls considerably short of the sorts of improvements seen in rat allografts and the more successful human allograft studies. Thus, we suggest that it is important to continue exploring alternative donor cell sources and recently we have begun again to consider the potential of short-term expanded hFNPs as donor cells for regenerative medicine. These cells may have the capacity to more readily respond to developmental signals that would allow them to maintain their identity over the period of expansion in culture. Such ‘epigenetic memory’ has been suggested from work in several adult somatic tissues (Hiler et al., 2015) and may be an important aspect of these cells and allow a more adaptable approach for generating large populations of MSNs for clinical application.

**Fig. 2:**
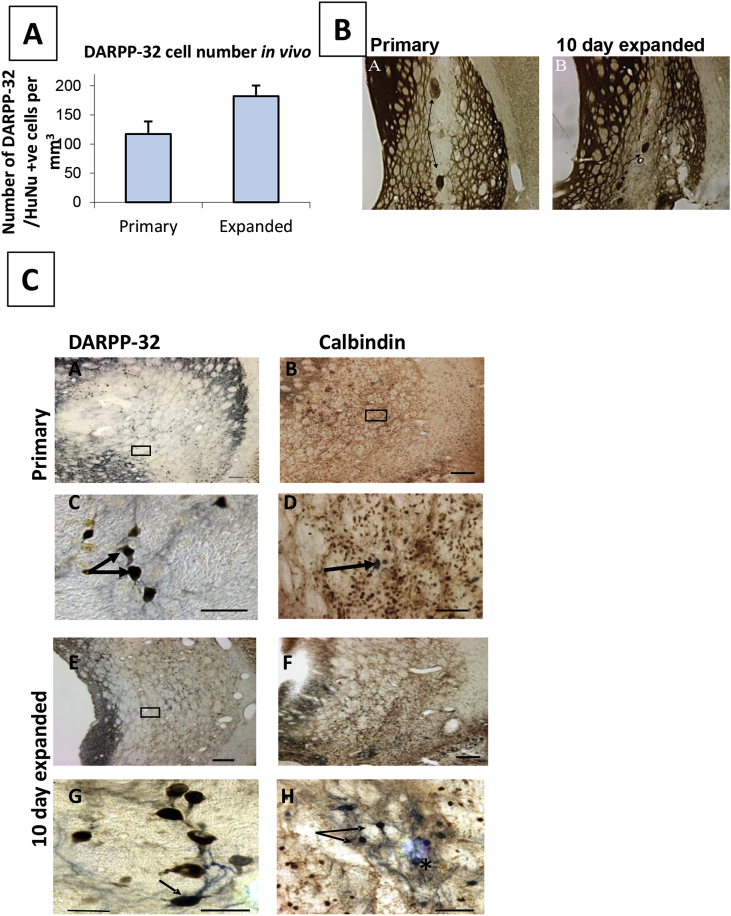
**Primary versus 10 day expanded WGE cells. A) Graph showing DARPP-32 counts *in vivo***. There was no significant difference between the number of DARPP-32/HuNu positive cells within the graft area in primary and 10 day expanded grafts. **B) Photomicrograph of Primary and expanded FNP AChE stained sections**. AChE is used as a marker of striatal-like “P zones” (arrows in A). Primary WGE grafts show organisation in to P and non-P zones, as expected, in contrast to expanded FNP graft which have fewer P zones and display a more homogeneous structure. **C) Photomicrographs of sections from Primary and 10 day expanded hFNPs transplanted to the rodent lesioned striatum**. Primary grafts contained DARPP-32/HuNu positive cells (A) and (C; higher power of A), as well as calbindin/HuNu positive cells (B) and arrow in (D; higher power of B). Expanded hFNP grafts also contained DARPP-32/HuNu positive cells (E) and (G; higher power of E), and Calbindin/HuNu positive cells (F) and (H; higher power of F) (arrows in H). Not all calbindin cells within the graft area were HuNu positive (asterisk in H). Scale bar = 100 μm. *Abbreviations:* AChE – acetylcholinesterase; HuNu – human nuclear antigen.

In a comparison of hFNP and hWGE grafts 20 weeks post-transplantation, graft-derived DARPP32 positive cells (a classic marker of MSNs) were seen both in primary WGE and expanded WGE grafts (Fig. 2), and stereological analysis revealed no significant difference in the number of graft-related DARPP-32- positive neurons (Fig. 2A). Both primary and expanded FNP grafts were also positive for calbindin (stains both MSN and striatal interneurons). Interestingly, hFNP grafts differed from hWGE grafts in an important aspects of morphological structure; WGE grafts are typically organised into “P zones” (containing mostly striatal-like cells), which stain for a variety of markers, including acetylcholinesterase (AChE), and “non P zones” containing mostly non-striatal (probably cortical-like) cells (Graybiel et al., 1989, Wictorin, 1992). The hFNP grafts appeared to show very weak, or no organisation into such zones, as shown by AChE staining (Fig. 2B). Our own interpretation of this is that the hFNP grafts may present an advantage in their lack of non-P zones; non-P zones are thought to result from cortical progenitors which migrate through the WGE during development and thus may ‘dilute’ the effect of the graft. However, it is important to emphasise that this is speculation and further studies are needed to resolve this issue.

To date, there is very limited literature reporting on functional assessments of hFNP grafts in HD models. McBride et al in 2004 reported improved functional effect on the cylinder task following transplantation of human cortical derived FNPs in the quinolinic acid lesion model of HD (McBride et al., 2004). However, this was not supported by histological evidence of graft-derived MSNs, based on DARPP-32 expression 8 weeks post transplantation. This relatively short time course for graft maturation may be the reason for the lack of DARPP-32 expression along with the fact that the cells were derived from the developing cortex, rather than WGE, and were expanded in culture for a long period of time, which as we have shown previously is not supportive to good graft survival (Zietlow et al., 2005, 2012). Studies using human fetal tissue-derived neural precursor immortalised cell lines have also reported survival, differentiation and integration of transplanted cells with modest behavioural recovery in some cases (for example Roberts et al., 2006, Ryu et al., 2004). However, it is clear that systematic studies examining the functional effects of WGE-derived FNPs in HD models are required.

In summary, there is some evidence that hFNPs retain the ability to default to an MSN-like phenotype following modest expansion *in vitro,* and also that they are capable of projecting to target specific brain regions. It is important to acknowledge that most studies to date, including the ones presented here, have relied on default neuronal differentiation of the transplanted FNPs post-transplantation. That is, FNPs are precursor cells, and in these experiments they have not undergone directed differentiation towards specific neuronal phenotypes. An important next step will be to subject FNPs to molecules known to be important for MSN differentiation.

## 4 Concluding remarks

In conclusion, we review the potential of two major sources of fetal-derived stem cells as donor cells for regenerative medicine in HD. At this stage in the development of CNS regenerative medicine it is important to properly consider all possible donor cell sources, as there are factors associated with each that make them more or less suitable for individual conditions. For example, another strategy is the use of fetal-derived iPSCs, on the basis that they may possess a different range of properties to adult-derived iPSCs, but there is currently very little literature in this area. Here we discuss our extensive attempts to produce hEG cells, which we were unable to convincingly generate and maintain, and this is consistent with the small numbers of reports of successful conversion in the literature. Further exploration of this cell type will need to await a better understanding of the biology of hPGCs. In contrast, we suggest that FNPs hold more promise. Although they cannot as yet be regarded as potential donor cells for neural transplantation, we suggest that there is sufficient positive data to support further exploration of their potential.
